# Supplementary material for: Reducing Panic Buying During Crisis Lockdowns: A Randomized Controlled Trial of a Theory-Based Online Intervention
Source: Behav Sci (Basel). 2025 Dec 24;16(1):42. doi: 10.3390/bs16010042 (PMC12837318; doi:10.3390/bs16010042)
Supplement: Supplementary file 1 [file behavsci-16-00042-s001.zip › behavsci-3766212-supplementary.pdf]

Supplementary Materials for "Reducing Panic Buying: A Randomised Controlled Trial of a Brief Online Video Intervention"

Figure S1. *Consort flow diagram.*

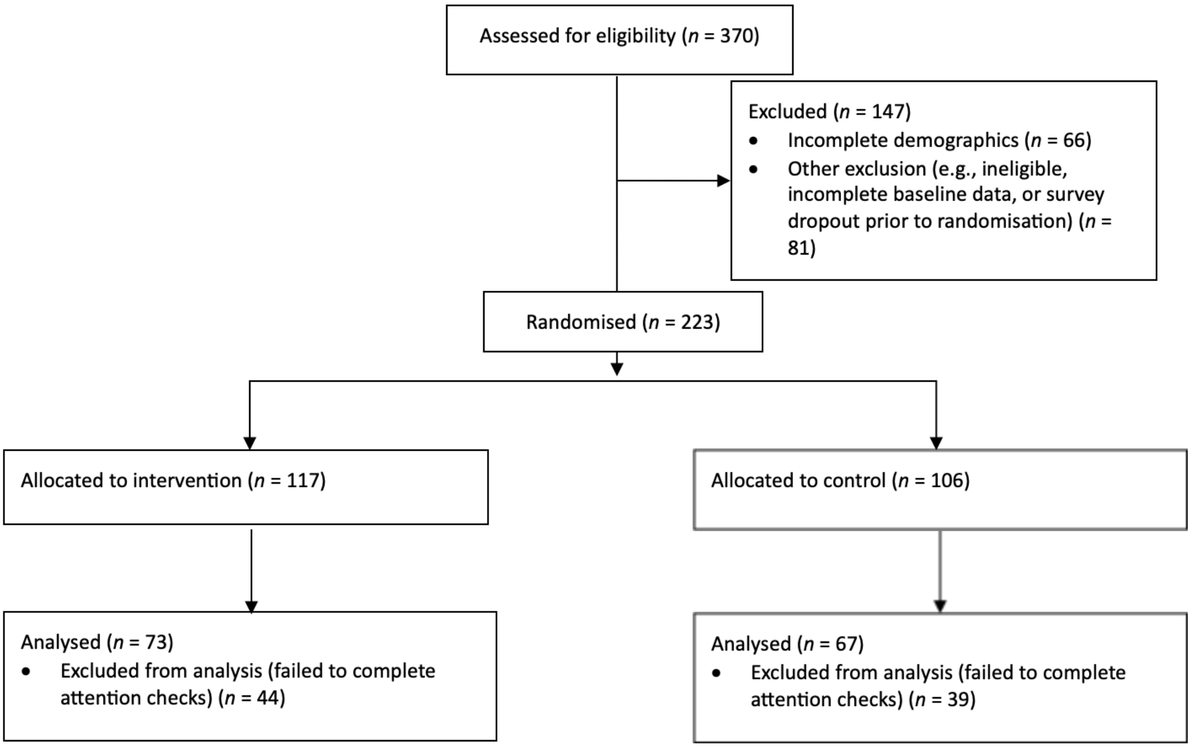

Table S1. *Survey Measures and Response Scales.*

Construct	Example Item(s)	Response Scale
Behaviour Measures	The following questions will ask you about the extent to which you have bought more products than you would use based on your usual frequency of shopping, since the COVID-19 pandemic began (approximately January 2020). The COVID-19 pandemic refers to the international outbreak of a severe acute respiratory syndrome caused by a new strain of coronavirus. We are interested in the following categories of products:<br>1. Non-perishable foods (e.g., pastas, rices, drinks, canned, flour, sugar, frozen vegetables)<br>2. Cleaning products (e.g., hand sanitiser, bleach, wipes, disinfectant, washing powder)<br>3. Hygiene products (e.g., toilet paper, tissues, nappies, nappy wipes, etc.)	*Please select the response that best characterises your buying behaviour in relation to [product category], since the COVID-19 pandemic began:*<br>• I have bought only the amount of [product category] that I usually buy<br>• I have increased my purchasing to buy enough [product category] for an extra few days<br>• I have increased my purchasing to buy enough [product category] for an extra week<br>• I have increased my purchasing to buy enough [product category] for an extra two weeks<br>• I have increased my purchasing to buy enough [product category] for an extra three weeks<br>• I have increased my purchasing to buy [product category] for an extra month<br>• I have increased my purchasing to buy [product category] for more than an extra month
Social-Cognition Factors	Consider the following scenario:<br>*It has just been announced by your state premier that your state will be re-entering lockdown in a few days due to a new outbreak of COVID-19. This means that you can only leave your house for essential reasons such as going to work or buying essential items. It is not yet known how long the lockdown will be imposed for. The media has also started to report that people have been "panic buying" and that supermarkets and grocery stores are running out of hygiene products such as toilet paper, non-perishable foods such as pasta and tinned vegetables, and cleaning products such as wipes and hand sanitiser.*<br><br>Now consider your future shopping behaviour, if such a scenario occurred, how likely are you to stock up on groceries…?<br><br>Each of the following measures will be administered with each of the three behaviours inserted.	
Willingness	• I would be [rate] to *<insert behaviour>* in this situation<br>• How likely is it that you would *<insert behaviour>* in this situation?<br>• In this situation would you plan to *<insert behaviour>*?	1 = Not willing, 7 = Willing<br><br>1 = Not at all likely, 7 = Very likely<br><br>1 = Definitely not, 7 = Definitely would

Planning	In this situation would you plan to <*insert behaviour*>?	1 = Definitely not, 2, 3, 4, 5, 6, 7 = Definitely would
Intentions	Do you agree that…? (Please indicate ONE option per line)<br>• I intend to <*insert behaviour*>.<br>• I expect I would <*insert behaviour*>.<br>• It is likely that I will <*insert behaviour*>.<br>• In general, I would be willing to <*insert behaviour*>.	1 = Strongly disagree,<br>2 = Disagree,<br>3 = Somewhat disagree,<br>4 = Neither agree nor disagree,<br>5 = Somewhat agree,<br>6 = Agree,<br>7 = Strongly agree
Attitudes	If I were to <*insert behaviour*>, it would be:<br>• Bad – Good<br>• Harmful – Harmless<br>• Unwise – Wise	1 = Bad, 7 = Good<br>1 = Harmful, 7 = Harmless<br>1 = Unwise, 7 = Wise
Subjective Norms	Do you agree that…? (Please indicate ONE option per line)<br>• Most people who are important to me would approve of me <*insert behaviour*>.<br>• Those people who are important to me think that I should <*insert behaviour*>.<br>• Those people who are important to me would want me to <*insert behaviour*>.<br>• Those people who are similar to me would <*insert behaviour*>.<br>• Most people like me would <*insert behaviour*>.	1 = Strongly disagree,<br>2 = Disagree,<br>3 = Somewhat disagree,<br>4 = Neither agree nor disagree,<br>5 = Somewhat agree,<br>6 = Agree,<br>7 = Strongly agree
Risk perception	Do you agree that…? (Please indicate ONE option per line)<br>• It would be risky for me not to <*insert behaviour*>.<br>• If I do not <*insert behaviour*>, there would be risk involved.	1 = Strongly disagree,<br>2 = Disagree,<br>3 = Somewhat disagree,<br>4 = Neither agree nor disagree,<br>5 = Somewhat agree,<br>6 = Agree,<br>7 = Strongly agree

Note. All items were administered for hypothetical purchasing of each of the three product categories: non-perishable foods, cleaning products, and hygiene products.

Table S2. *Cronbach's alpha values for all multi-item measures across product categories.*

Construct	Non-perishable	Hygiene	Cleaning
Willingness	.87	.91	.85
Intention	.90	.90	.86
Attitudes	.85	.82	.81
Subjective Norms	.90	.91	.91
Risk Perception	.90	.90	.88

Table S3. *Descriptive statistics and estimated marginal means (EMMs) for primary outcomes (willingness and intention) across product categories (non-perishable, cleaning, hygiene) by group (intervention, control) and timepoint (pre-intervention, post-intervention). Values shown include sample size (N), raw means, standard deviations (SD), and EMMs with standard errors (SE) and 95% confidence intervals (CI).*

Outcome	Product Type	Group	Time	N	Raw Mean	SD	EMM	SE	95% CI Lower	95% CI Upper
Willingness	Non-perishable	Control	T1	67	3.85	1.57	3.85	0.20	3.45	4.25
		Control	T2	67	3.93	1.85	3.93	0.21	3.50	4.35
		Intervention	T1	73	3.66	1.73	3.66	0.19	3.28	4.05
		Intervention	T2	73	3.26	1.66	3.26	0.21	2.85	3.66
Intention	Non-perishable	Control	T1	67	3.29	1.73	3.29	0.21	2.87	3.71
		Control	T2	67	3.16	1.82	3.16	0.21	2.75	3.57
		Intervention	T1	73	3.01	1.75	3.01	0.20	2.60	3.41
		Intervention	T2	73	2.39	1.57	2.39	0.20	2.00	2.79
Willingness	Cleaning	Control	T1	67	3.10	1.39	3.10	0.18	2.74	3.47
		Control	T2	67	3.30	1.59	3.30	0.19	2.92	3.67
		Intervention	T1	73	3.20	1.61	3.20	0.18	2.85	3.55
		Intervention	T2	73	2.97	1.52	2.97	0.18	2.61	3.33
Intention	Cleaning	Control	T1	67	2.49	1.54	2.49	0.19	2.11	2.87
		Control	T2	67	2.59	1.68	2.59	0.19	2.23	2.96
		Intervention	T1	73	2.40	1.61	2.40	0.18	2.04	2.77
		Intervention	T2	73	2.04	1.37	2.04	0.19	1.69	2.39
Willingness	Hygiene	Control	T1	67	3.57	1.74	3.57	0.23	3.12	4.01
		Control	T2	67	3.70	1.75	3.70	0.21	3.28	4.12
		Intervention	T1	73	3.75	1.93	3.75	0.22	3.32	4.18
		Intervention	T2	73	3.25	1.71	3.25	0.20	2.85	3.65
Intention	Hygiene	Control	T1	67	2.87	1.70	2.87	0.21	2.45	3.29
		Control	T2	67	2.90	1.80	2.90	0.21	2.49	3.32
		Intervention	T1	73	2.84	1.77	2.84	0.20	2.44	3.24
		Intervention	T2	73	2.38	1.65	2.38	0.20	1.98	2.78

Table S4. *Descriptive statistics and estimated marginal means (EMMs) for secondary outcomes (attitudes, subjective norms, and risk perception) across product categories (non-perishable, cleaning, hygiene) by group (intervention, control) and timepoint (pre-intervention, post-intervention). Values shown include sample size (N), raw means, standard deviations (SD), and EMMs with standard errors (SE) and 95% confidence intervals (CI).*

Outcome	Product Type	Group	Time	N	Raw Mean	SD	EMM	SE	95% CI Lower	95% CI Upper
Attitudes	Non-perishable	Control	T1	67	2.98	1.66	2.98	0.20	2.58	3.38
		Control	T2	67	2.84	1.66	2.84	0.19	2.46	3.22
		Intervention	T1	73	3.08	1.68	3.08	0.20	2.69	3.46
		Intervention	T2	73	2.16	1.49	2.16	0.18	1.80	2.53
Subjective Norms	Non-perishable	Control	T1	67	3.67	1.35	3.68	0.19	3.30	4.05
		Control	T2	67	3.62	1.50	3.62	0.20	3.23	4.01
		Intervention	T1	73	3.40	1.69	3.40	0.18	3.04	3.76
		Intervention	T2	73	2.97	1.69	2.97	0.19	2.59	3.34
Risk Perception	Non-perishable	Control	T1	67	3.53	1.84	3.53	0.22	3.10	3.96
		Control	T2	67	3.39	1.85	3.39	0.23	2.94	3.83
		Intervention	T1	73	3.37	1.72	3.37	0.21	2.96	3.78
		Intervention	T2	73	2.82	1.86	2.82	0.22	2.39	3.25
Attitudes	Cleaning	Control	T1	67	2.72	1.69	2.72	0.23	2.25	3.18
		Control	T2	67	2.70	1.75	2.70	0.20	2.31	3.10
		Intervention	T1	73	3.23	2.11	3.23	0.23	2.79	3.68
		Intervention	T2	73	2.10	1.51	2.10	0.19	1.73	2.48
Subjective Norms	Cleaning	Control	T1	67	3.38	1.36	3.38	0.19	3.01	3.75
		Control	T2	67	3.42	1.52	3.42	0.19	3.04	3.80
		Intervention	T1	73	3.37	1.66	3.37	0.18	3.02	3.73
		Intervention	T2	73	2.85	1.63	2.85	0.19	2.49	3.22
Risk Perception	Cleaning	Control	T1	67	2.77	1.58	2.77	0.20	2.38	3.16
		Control	T2	67	2.80	1.69	2.80	0.20	2.41	3.20
		Intervention	T1	73	2.76	1.62	2.76	0.19	2.39	3.13
		Intervention	T2	73	2.38	1.57	2.38	0.19	2.00	2.75
Attitudes	Hygiene	Control	T1	67	2.78	1.66	2.78	0.23	2.32	3.24
		Control	T2	67	2.77	1.73	2.77	0.20	2.37	3.17
		Intervention	T1	73	3.24	2.08	3.24	0.22	2.80	3.67
		Intervention	T2	73	2.16	1.56	2.16	0.19	1.78	2.54
Subjective Norms	Hygiene	Control	T1	67	3.65	1.57	3.65	0.20	3.25	4.05
		Control	T2	67	3.70	1.62	3.70	0.21	3.28	4.12
		Intervention	T1	73	3.60	1.73	3.60	0.19	3.22	3.98
		Intervention	T2	73	3.03	1.84	3.03	0.20	2.63	3.43
Risk Perception	Hygiene	Control	T1	67	3.16	1.70	3.16	0.22	2.73	3.60
		Control	T2	67	3.13	1.84	3.13	0.24	2.66	3.59
		Intervention	T1	73	3.37	1.91	3.37	0.21	2.95	3.79
		Intervention	T2	73	2.80	1.93	2.80	0.23	2.35	3.24
